# Dietary composition and spatial patterns of polar bear foraging on land in western Hudson Bay

**DOI:** 10.1186/1472-6785-13-51

**Published:** 2013-12-21

**Authors:** Linda J Gormezano, Robert F Rockwell

**Affiliations:** 1 Division of Vertebrate Zoology, American Museum of Natural History, 79th Street and Central Park West, 10024 New York, NY, USA

## Abstract

**Background:**

Flexible foraging strategies, such as prey switching, omnivory and food mixing, are key to surviving in a labile and changing environment. Polar bears (*Ursus maritimus)* in western Hudson Bay are versatile predators that use all of these strategies as they seasonally exploit resources across trophic levels. Climate warming is reducing availability of their ice habitat, especially in spring when polar bears gain most of their annual fat reserves by consuming seal pups before coming ashore in summer. How polar bears combine these flexible foraging strategies to obtain and utilize terrestrial food will become increasingly important in compensating for energy deficits from lost seal hunting opportunities. We evaluated patterns in the composition of foods in scat to characterize the foraging behaviors that underpin the diet mixing and omnivory observed in polar bears on land in western Hudson Bay. Specifically, we measured diet richness, proportions of plant and animal foods, patterns in co-occurrence of foods, spatial composition and an index of temporal composition.

**Results:**

Scats contained between 1 and 6 foods, with an average of 2.11 (*SE* = 0.04). Most scats (84.9%) contained at least one type of plant, but animals (35.4% of scats) and both plants and animals occurring together (34.4% of scats) were also common. Certain foods, such as Lyme grass seed heads (*Leymus arenarius)*, berries and marine algae, were consumed in relatively higher proportions, sometimes to the exclusion of others, both where and when they occurred most abundantly. The predominance of localized vegetation in scats suggests little movement among habitat types between feeding sessions. Unlike the case for plants, no spatial patterns were found for animal remains, likely due the animals’ more vagile and ubiquitous distribution.

**Conclusions:**

Our results suggest that polar bears are foraging opportunistically in a manner consistent with maximizing intake while minimizing energy expenditure associated with movement. The frequent mixing of plant-based carbohydrate and animal-based protein could suggest use of a strategy that other Ursids employ to maximize weight gain. Further, consuming high rates of certain vegetation and land-based animals that may yield immediate energetic gains could, instead, provide other benefits such as fulfilling vitamin/mineral requirements, diluting toxins and assessing new foods for potential switching.

## Background

Flexibility in foraging is key to persisting in a labile and changing environment (e.g., [1-4]). Three common strategies are used by predators to exploit available food in such situations: prey switching, omnivory and food mixing [5-7]. Prey switching involves shifting between ecologically diverse prey, seasonally or over an animal’s lifetime in response to the availability or quality of the prey [2,7,8]. Omnivory is defined as foraging on both animal and plant material and can benefit species that are primarily carnivorous by providing an alternate source of nutrition when preferred animal-based food is in short supply or not easily obtained [6,9,10]. Food mixing involves ingesting material from different species either simultaneously or over various intervals of an animal’s lifetime that differ qualitatively to the consumer [11]. For example, brown bears (*Ursus arctos*) and Speck’s hinge back tortoises (*Kinixys spekii*) consume specific combinations of different foods to obtain optimal proportions of macronutrients [6,12].

Polar bears (*Ursus maritimus*), especially those that spend portions of the year on land, are versatile predators and appear to use all of these strategies, as they seasonally exploit food across trophic levels (e.g., [13-16]). Although the more southern populations of Hudson Bay are pagophilic (ice-loving) for much of the year, they move to land for a minimum of 4–5 months as the sea ice melts completely by summer [17]. While on the sea ice, they are mostly carnivorous, feeding primarily on ringed seals (*Phoca hispida*) but periodically consuming bearded seals (*Erignathus barbatus*), harbor seals (*Phoca vitulina*) and other marine mammals (e.g., [18]). As these “southern” polar bears move to land, they adopt a more omnivorous and mixed diet including fruit and other vegetation as well as different varieties of animals (e.g., [14,16,19]).

Climate change is causing Hudson Bay sea ice to melt earlier in the spring and this increasingly limits the time polar bears have to hunt seal pups, from which they historically have gained the majority of their annual fat reserves [17]. These reduced hunting opportunities are believed to have resulted in nutritional deficits that have been linked to decreases in survival and reproductive output of some demographic groups [20,21]. Ironically, the earlier melting of sea ice that has resulted in a mismatch with their traditional spring prey has also produced a new match with land-based prey on the Cape Churchill Peninsula of western Hudson Bay [22]. Earlier onshore arriving polar bears are now taking advantage of lesser snow geese (*Chen caerulescens caerulescens*) and their eggs as well as caribou (*Rangifer tarandus*) from the increasing populations of both species [16,23].

It is possible that, once ashore, switching to these new land-based prey could offset some of the nutritional deficits incurred by earlier arriving polar bears and mitigate some of the reductions in survival and reproductive success [16,22,23]. It is more likely, however, that those deficits could be offset if these resources are combined with other readily available plant and animal land-based resources these polar bears consume during the ice-free period [23,24]. Such food mixing and omnivory can result in synergisms that lead to otherwise unexpected nutritional gains (e.g., [12]). Unfortunately, little is known about the basic foraging patterns that might underpin omnivory and food mixing in polar bears during the ice-free period [24] and that behavioral perspective is crucial to understanding the potential utility of these strategies [11]. For example, what foods are consumed over similar time frames and how is that consumption related to the spatial distribution of those foods?

The range of terrestrial foods sought by polar bears suggests a high level of plasticity in their foraging behavior which may have always been present (e.g., [25]), but actually might be increasing over time in response to changing ecological conditions [26]. For example, polar bears have been observed chasing and capturing lesser snow geese on land [27], climbing rocky outcrops to eat thick-billed murres (*Uria lomvia*) and their eggs [28], leaving the ice to consume eggs on land [29,30] and traveling to land or further inland to consume lower quality vegetation (compared to animals) such as graminoids and berries [31,32]. Again, however, what is not well known is how regularly these foods occur together in the diet of polar bears, especially during the ice-free period when the benefits of omnivory and food mixing could offset nutritional deficits [24].

In this paper, we use data from a large-scale polar bear scat collection on the Cape Churchill Peninsula of western Hudson Bay to examine patterns in dietary composition and richness within and between feeding sessions (as defined by the foods present in a scat pile) and how these vary across the landscape to more fully understand the extent and potential utility of omnivory and food mixing behaviors on land. Specifically, we examine (1) *diet richness* to evaluate how many items polar bears generally consume within foraging sessions; (2) *food-specific co-occurrence* to see if certain foods are consumed with fewer accompanying foods compared to other items; (3) *degree of omnivory* to determine to what degree polar bears are consuming different food types (e.g., vegetation, animals) alone or in combinations; (4) *spatial composition* to see if polar bears are depositing scats (and likely consuming foods) where they are most available; and (5) *spatial food-specific co-occurrence* to see if polar bears consume fewer accompanying foods when consuming certain foods that occur relatively more frequently in scats in a particular area. In addition, we use a rough temporal index to compare composition and food-specific co-occurrence rates in scats collected fresh in mid-summer compared to older “unknown” age scats to examine foraging patterns limited to that time period.

## Methods

### Study area

Scat was collected along 160 km of coastline and inland areas within the Cape Churchill Peninsula [22] where polar bears are known to occur during the ice-free period in Manitoba, Canada [33]. The sampling area extended from the town of Churchill, Manitoba (58°46′N, 94°12′W), east to Cape Churchill (58°47′N, 93°15′W) and south to Rupert Creek (57°50′N, 92°44′W). Samples collected from 6 denning areas southeast of Churchill extended inland of the coastline to 93°51′W (Additional file 1: Figure S1). For site description details, see Gormezano and Rockwell [16]. During the sampling period, polar bears were predicted to arrive on land shortly after 24 June, 22 June and 28 June in 2006, 2007 and 2008, respectively, based on standard calculations for 50% sea ice breakup [34,35].

### Scat collection and analysis

We used a trained detection dog to find scats along 1–3 kilometer linear coastal transects (parallel to the coastline) and in the vicinity of inland dens from 2006 through 2008. Coastal transects from the town of Churchill and Rupert Creek were walked between 25 May and 11 August. Upland habitat in the vicinity of inland dens was searched between 30 May and 17 June when they were likely to be vacant. For all scats collected, we recorded the date, geographic coordinates, substrate and relative freshness. Samples were categorized as either “fresh” (from the current season) or “unknown age” (from the current or previous season) based on smell, color and presence of insect larvae. Intact scats of all ages were collected and foods were identified from entire piles. Samples collected prior to the arrival of polar bears in a given year (see above) were all from a previous season (old) whereas those collected after that date were a mixture of fresh and old scats. Because freshness of scats was dependent, in part, on time of collection, we use the composition of “fresh” samples collected after the bears’ arrival only to identify foods definitely consumed in mid to late summer (and not previously). All collection protocols were approved by the Institutional Animal Care and Use Committee of the American Museum of Natural History (Reference Number: 11-1025-2005).

Scats were often found to be clumped along a transect line or near a denning site. To minimize potential bias resulting from multiple scats being deposited by a single individual, we did not use all of the samples collected from clumped points along each of our 31 transects for these diet analyses. We randomly selected approximately 50% of the scats collected from each transect for analysis so that they would be representative of the relative frequencies and geographic extent of the sampled areas. Though the actual number of polar bears depositing the sampled scats is unknown, we assume from the size and geographic extent of our sampling and other studies suggesting that polar bears segregate and move little once ashore [33] that our samples are representative of the land-based diet of those polar bears that do forage on the Cape Churchill Peninsula.

Animal remains were identified from entire scats using a combination of microscopy, reference keys [36-39] and expert opinion (N. Duncan, A. Rodriguez, C. Dove). Plant and fungi were identified using keys [40,41] but most were subsequently pooled into broad taxonomic categories due to the variety encountered and time constraints. Identification techniques are described in detail in Gormezano and Rockwell [16]. Bones, hairs and feathers were identified to the lowest taxonomic level possible but if they could not be identified beyond ‘bird’ or ‘mammal’ they were only included in statistical analyses where pooled, higher taxonomic groups (i.e., birds, mammals) were used. Bones classified no finer than ‘animal’ were only included in summary statistics of major food categories (e.g., vegetation, animals).

Polar bear hair was found in most scats and was likely ingested during grooming. We distinguished evidence of cannibalism from grooming by the larger volume of hair, presence of flesh, bone and a distinct smell. All food items (other than polar bear) were considered present if they were identified in a scat pile, regardless of volume.

### Statistical analysis

We examined the diet of polar bears using the: (1) raw frequencies (number of times each food item was found) and (2) scat occurrences (the number of scats with a food item). We use the percentages of these (relative to their appropriate sum) for ease of presentation in some cases. Raw frequencies of individual food items were found to occur independently in scats, justifying their use in statistical analyses [16]. The raw frequencies and the scat occurrences are the same value unless multiple items from the same category occur in a scat pile (i.e., 2 birds in one scat). Multiple items were only counted for animals when evidence was conclusive (e.g., 3 bird feet) and was not counted for plants and fungi.

Most analyses of spatial and compositional patterns in diet were done using 14 inclusive groups of food items with each group having at least 5 occurrences of all included taxa. These groups were polar bear, seal (e.g., *Phoca hispida)*, caribou (*Rangifer tarandus*), rodents (i.e., muskrats (*Ondatra zibethicus*), meadow voles (*Microtus pennsylvanicus*), collared or bog lemmings (*Dicrostonyx richardsoni* and *Synaptomys cooperi*)), birds, eggs, Lyme grass (*Leymus arenarius;* shafts and/or seed heads), Lyme grass seed heads (seed heads only), other grasses (e.g., *Festuca brachyphylla)*, marine algae (e.g., *Fucus* spp., *Laminaria* spp.), berries (e.g., *Vaccinium uliginosum*, *Empetrum nigrum*), mosses (e.g., *Sphagnum fuscum*), mushrooms (*Lycoperdon* and *Bovista* spp.) and garbage. Although the Lyme grass seed heads and shafts come from the same plant, their raw frequencies within scats are independent and they are treated as separate food items [16].

We tabulated the percent scat occurrences that included at least one food item that was: vegetation, animal (mammals, birds or eggs) and land-based animal (*LBA*; i.e., birds, eggs, caribou, rodents) across all piles. As an index of the complexity of the diet of individual bears, we also calculated the number of scats containing both vegetation and animal, >1 animal and >1 *LBA*.

As an additional index of diet complexity, we calculated the minimum, maximum and mean number of food types per pile using scat occurrences as the unit of measure. Because the “Lyme grass” category includes both the shafts and/or seed heads, including “Lyme grass seed heads” as a separate category when scat occurrences are the unit of measure is redundant. For this reason, “Lyme grass seed heads” was excluded from this analysis (13 groups used). To examine whether complexity differed depending on the presence of a particular food type, we quantified the scat occurrences of co-occurring food items in each scat for each of the 14 food categories and plotted their distribution and mean (with standard error) across all scats. Different animal and plant matter pass through the digestive tract of bears at different rates [42], so we assumed that the observed combinations reflect foods consumed within a single feeding session, not necessarily at the same time. We define a ‘feeding session’ as the period between ingesting and defecating the undigested remains, which can vary between 6.2 and 19.0 hours based on minimum digestive rates for vegetation (by grizzly bears) [43] and maximum digestion rates for meat (by polar bears) [44], respectively.

To examine potential effects of spatial differences in topography, vegetation and local prey abundance that might affect diet composition, raw frequencies of different food items were compared across 5 different sections of the study area. Although polar bears are capable of traversing long distances, they are known to move relatively little on land compared to on the ice [33,45]. We therefore hypothesized that scats collected from areas with distinct landscape characteristics, such as anthropogenic land use (e.g., the town of Churchill, tundra vehicle based tourism), concentrations of known nesting bird colonies, and distinct vegetation clines [46], would contain food items specific to the areas from which they were collected. For example, we expected to see more garbage where people reside, more berries inland and more birds in scats in the vicinity of the historical lesser snow goose (*Anser caerulescens caerulescens*, henceforth snow goose) and common eider (*Somateria mollissima*) colonies near La Pérouse Bay.

Using the raw frequencies of items from the 14 inclusive food groups, we pooled items to major categories (animal, vegetation or garbage). To evaluate if there was an overall difference in the proportions of these categories among areas, we used a 5 × 3 log-likelihood chi-square test. For this test, the 3 food categories were cross-classified against the 5 areas and expectations computed under the independence assumption as the product of the proportion of scats containing the food category and the proportion of scats in the area times the total number of scats. The log-likelihood chi-square was used rather than the chi-square because it is less affected by low cell frequencies [47]. Expectations for subsequent log-likelihood chi-square analyses were computed in a similar fashion. Because this overall test was significant (*G* = 100.27, *DF* = 8, *P* < 0.0001), indicating a difference in proportions, we performed 5 × 2 log-likelihood chi-square tests for each food category to identify in which category items varied.

We performed a similar test evaluating differences in the proportions of individual foods (from the 14 inclusive groups) across the study area using a 5 × 14 log-likelihood chi-square test. Because the overall test was highly significant (*G* = 376.14, *DF* = 52, *P* < 0.0001), indicating differences among the 5 sections of the study area, we performed 2 × 14 log-likelihood chi-square tests for each of the 5 sections to identify which had food items that varied. Significance of these pair-wise tests was evaluated using a sequential Bonferroni approach [48] to reduce inflation of our overall α–error rate. For the sections in which items differed, we then compared the proportions and 95% confidence limits of the frequencies of each food item to identify which ones differed the most.

To test the hypothesis that polar bears would consume certain foods more frequently in a particular area to the exclusion of others, whether because of preference or availability, we compared the means and 95% confidence intervals of scat occurrences of co-occurring foods in areas where foods were consumed more frequently with those from all other areas. We hypothesized that if other foods were being excluded the mean number of co-occurring foods in those areas would be less than (and outside the confidence interval of) all other areas. We illustrate our results by plotting the differences between mean number of co-occurring items among scats containing foods consumed relatively more often in a particular area and the mean number of co-occurring foods in scats containing these same items in all other areas. Because we use mean differences, a value of zero equals no difference. Pooled estimates of variance are used in derivation of confidence limits [47].

Although we could not assign exact age to most scats, it was possible to identify those deposited in the current season. Because our sampling occurred just as polar bears were arriving ashore, we assumed that these scats contained foods consumed either on the ice (just before coming ashore) or shortly after arriving. Using raw frequencies as the unit of measure, we performed a 2 × 3 log-likelihood chi-square to evaluate whether there were differences in the proportions of major food categories (animals, vegetation and garbage) between fresh and unknown age scats. We then performed a 2 × 14 log-likelihood chi-square test to assess whether the frequencies of individual foods (within these broad categories) differed in fresh and unknown age scats. Because the overall test was highly significant (*G* = 36.79, *DF* = 13, *P* = 0.0004), indicating differences between foods in fresh and unknown age scats, we compared the proportions and 95% confidence limits of the frequencies of food items to identify which ones were being consumed and deposited in scat more or less often when polar bears first come ashore.

To evaluate whether polar bears were consuming certain foods at relatively higher rates to the exclusion of others in mid-summer, we also compared the mean number and 95% confidence interval of scat occurrences of co-occurring foods for new and unknown age scats with more frequently consumed items. Results are illustrated using differences and 95% confidence intervals of mean numbers of co-occurring items in fresh scats containing the more frequently consumed items and the mean numbers of co-occurring items in unknown age scats containing these same items.

## Results

We evaluated 642 scats (of 1,262 collected); 219, 248 and 175 in 2006, 2007 and 2008, respectively. 593 scats were collected from coastal areas and 49 from inland sites. Vegetation and land-based animals occurred in 84.9% and 35.4% of all scats, respectively. Polar bears that consumed animals (either land- or marine-based; 45.8% of scats) did not appear to specialize on that particular resource because we also observed a high co-occurrence of animal and vegetation (34.3%) and multiple animal taxa (9.3%) in the same scat (Table 1).

**Table 1:** The number and percentage of polar bear scats (n = 642) containing ‘vegetation’, ‘animal’, ‘land-based’ food items

		**Scats containing food item**	
		**#**	**%**
Food type (≥ 1)			
	Vegetation	545	84.9
	Animal	294	45.8
	Land-based food	605	94.2
	Land-based animal	227	35.4
Food combinations			
	Animal + Vegetation	220	34.3
	> 1 Animal	60	9.3
	> 1 Land-based animal	42	6.5

‘Vegetation’ includes grasses, marine algae, mosses, mushrooms and berries; ‘Animal’ includes identified and unidentified birds, mammals and eggs; ‘Land-based food’ includes any food item except seal or polar bear (which could have been consumed on the ice); ‘Land-based animal’ includes caribou, birds, eggs and rodents.

**Figure 1:**
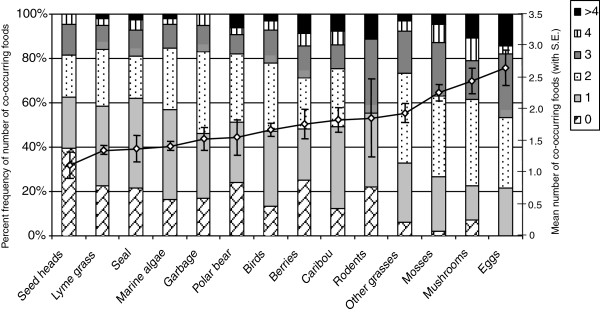
**The distribution of co-occurring foods in polar bear scats collected from western Hudson Bay from 2006–2008 as an index of diet complexity.** For each food item on the x-axis, each section of the vertical bars is the percent frequency of the number of co-occurring food items. For example, seed heads occurred alone in 39.5% of scats, with one other food item in 23.3% of scats, etc. The diamond points connected by the black line are the mean number of co-occurring foods (right y-axis) with associated standard errors for each food item.

There were between 1 and 6 different foods in each scat, with an average of 2.11 (*SE* = 0.04) items. The mean number of co-occurring items ranged from 1.21 (*SE* = 0.19) for Lyme grass seed heads to 2.61 (*SE* = 0.26) for eggs (Figure 1). The percentage of scats that were found with 0, 1, 2, 3, 4 and >4 accompanying items is also illustrated for each of the 14 food items in Figure 1.

The overall proportions of food categories (animals, vegetation and garbage) differed across the study area (*G* = 100.27, *DF* = 8, *P* < 0.0001), but this difference was only due to differences in the proportions of vegetation (*G* = 39.25, *DF* = 4, *P* < 0.0001) and garbage (*G* = 82.27, *DF* = 4, *P* < 0.0001). The proportions of animals (*G* = 11.14, *DF* = 4, *P* < 0.025) did not significantly differ at our adjusted alpha level (α = 0.0167). Individual food items significantly differed across the study area (*G* = 376.14, *DF* = 52, *P* < 0.0001). More specifically, area #1 (*G* = 95.62, *DF* = 13, *P* < 0.0001, *n* = 69 scats), area #2 (*G* = 55.45, *DF* = 13, *P* < 0.0001, *n* = 71), area #4 (*G* = 149.49, *DF* = 13, *P* < 0.0001, *n* = 369) and area #5 (*G* = 180.58, *DF* = 13, *P* < 0.0001, *n* = 49) each had food items that occurred in different proportions than expected given total occurrences in all other areas. The proportions for area #3 (*G* = 25.30, *DF* = 13, *P* = 0.021, *n* = 84) were not significantly different using our adjusted alpha level (α = 0.01).

**Figure 2:**
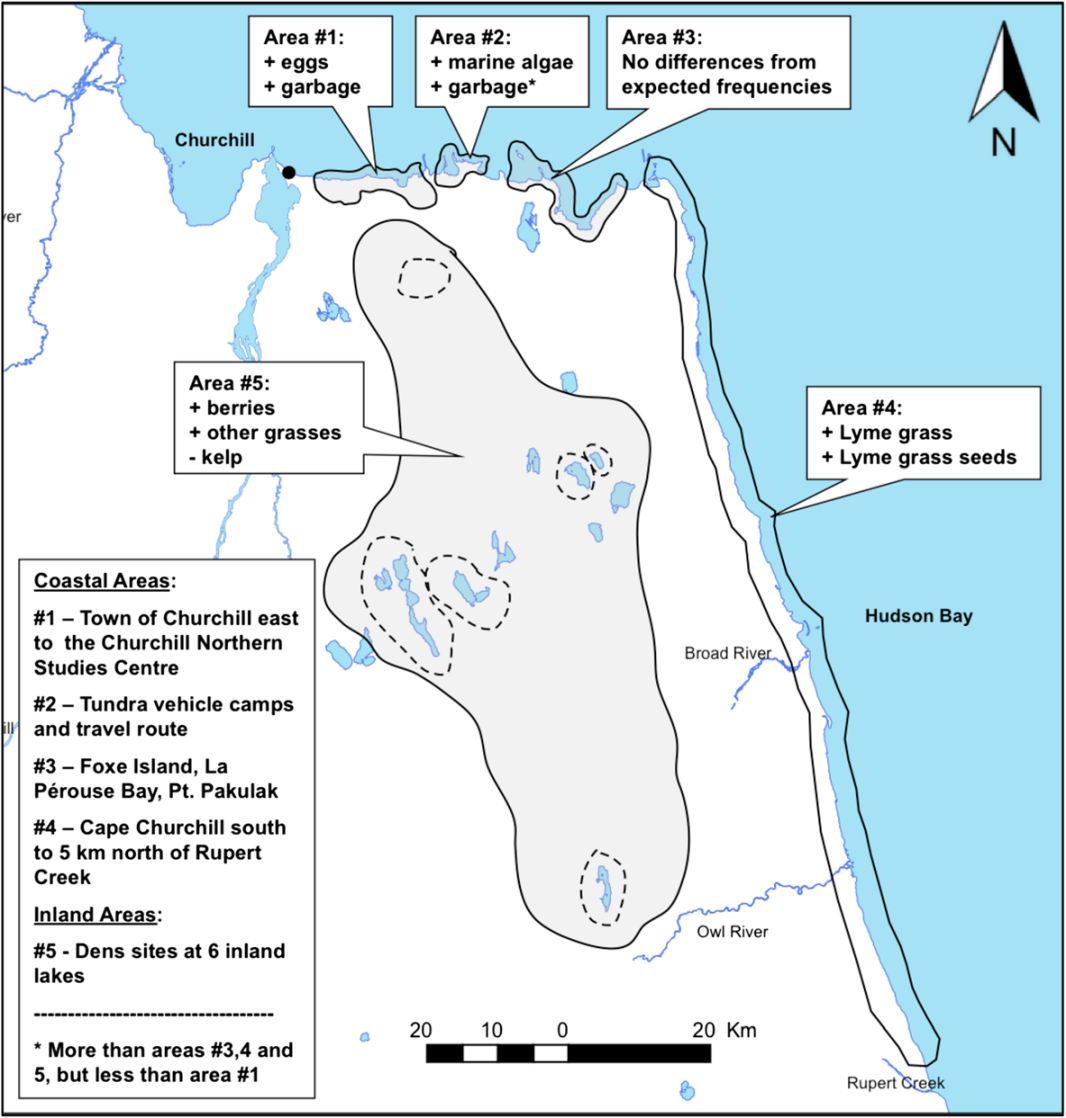
**Spatial differences in occurrences of food items from polar bear scats across the study area.** Our sampling area was divided into 5 sections based on anthropogenic land use, concentrations of known prey and vegetation clines. Classes of food items that occurred more (+) or less (-) often than expectations based on other areas are indicated.

Within and adjacent to the town of Churchill (area #1, Figure 2), we found scats with more eggs and garbage. Further east, along the tundra vehicle route, which runs between two temporary camps set up by tundra vehicle tour operators in the fall (area #2), we found a higher proportion of marine algae and garbage (more than areas #3, 4 and 5, but less than area #1). In the stretch of coast south of Cape Churchill to just north of Rupert Creek (area #4), we found a higher proportion of scats with Lyme grass shafts and Lyme grass seed heads. Inland areas near dens (area #5) had significantly more berries, other grasses and less marine algae. No significant differences in proportions were detected along the coast near La Pérouse Bay (area #3). Proportions of food item frequencies in each area with confidence limits are summarized in Table 2.

**Table 2:** The proportions and upper and lower 95% confidence limits of the frequencies of food items in 5 different areas across the study area

	**Site #**				
**Food item:**	**1**	**2**	**3**	**4**	**5**
**Birds**	20.43	19.96	27.46	13.26	24.70
	**14.01**	**12.98**	**20.97**	**10.89**	**15.79**
	9.00	9.07	15.35	8.84	9.09
					
	18.23	2.78	6.16	2.46	5.73
**Eggs**	12.98*	**0.00**	**2.69**	**1.38**	**1.05**
	9.52	0.00	0.88	0.69	0.03
					
	8.09	8.30	4.96	7.17	14.63
**Caribou**	**3.82**	**3.82**	**2.15**	**5.38**	**7.37**
	1.42	1.25	0.59	3.93	3.01
					
	7.25	1.49	4.64	4.45	5.73
**Polar bears**	**3.18**	**0.00**	**1.61**	**3.00**	**1.05**
	1.04	0.00	0.33	1.93	0.03
					
	6.36	3.10	2.96	1.28	3.81
**Rodents**	**2.55**	**0.76**	**0.54**	**0.50**	**0.00**
	0.70	0.02	0.01	0.14	0.00
					
	8.09	8.70	8.30	4.29	3.81
**Seals**	**3.82**	**3.82**	**4.30**	**2.88**	**0.00**
	1.42	1.25	1.87	1.83	0.00
					
	20.43	31.84	29.88	34.81	22.24
**Lyme grass**	**14.01**	**23.66**	**23.12**	32.04*	**13.68**
	9.00	16.71	17.28	31.03	7.51
					
	4.51	1.49	4.64	6.46	3.81
**Lyme grass seed heads**	**1.27**	**0.00**	**1.61**	4.76*	**0.00**
	0.15	0.00	0.33	3.38	0.00
					
	8.09	7.64	6.88	6.89	18.51
**Other grasses**	**3.82**	**3.05**	**3.23**	**5.13**	10.53*
	1.42	0.84	1.19	3.71	5.16
					
	26.16	47.10	32.15	23.39	7.39
**Marine algae**	**19.11**	38.17*	**25.27**	**20.78**	**2.11**
	13.31	29.76	19.21	18.08	0.26
					
	8.09	3.10	4.96	2.30	47.29
**Berries**	**3.82**	**0.76**	**2.15**	**1.25**	36.84*
	1.42	0.02	0.59	0.60	27.16
					
	7.25	3.10	9.66	6.61	5.73
**Mushrooms**	**3.18**	**0.76**	**5.38**	**4.88**	**1.05**
	1.04	0.02	2.60	3.49	0.03
					
	8.98	10.71	9.66	13.20	17.27
**Moss**	**4.46**	**5.34**	**5.38**	**6.76**	**9.47**
	1.81	1.77	2.60	5.12	4.42
					
	22.58	12.64	4.64	1.10	5.73
**Garbage**	15.92*	**6.87**	**1.61**	**0.38**	**1.05**
	10.59	4.82	0.33	0.08	0.03

Proportions (in bold) with confidence limits that do not overlap the proportions of another value are considered significantly different (*) from other values.

**Figure 3:**
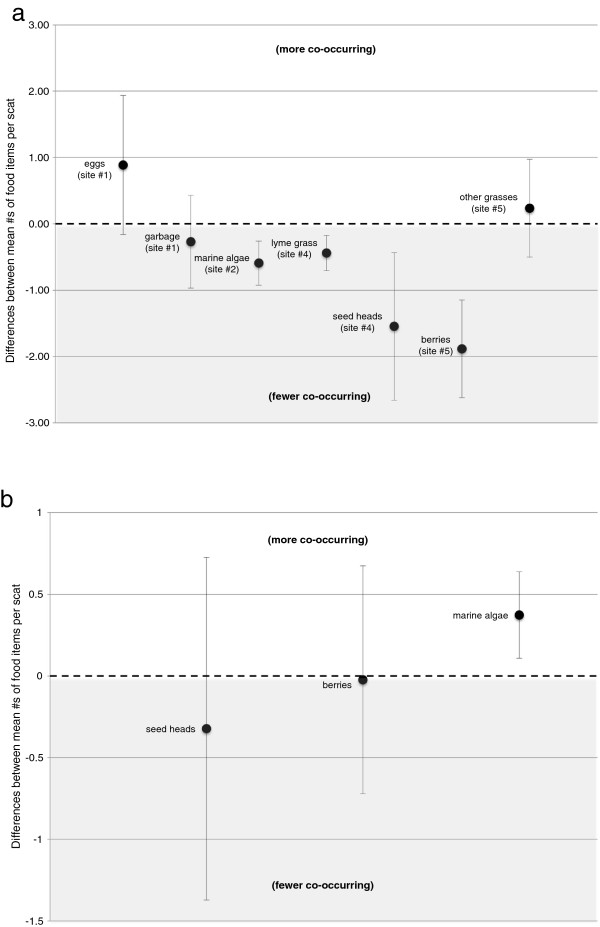
**Foods consumed at relatively higher rates by polar bears to the exclusion of other foods (a) across the study area and (b) between fresh and unknown age scats.** Black dots in **(a)** represent the differences between mean number of items co-occurring with foods consumed more often in a particular area and the mean number of co-occurring foods with these same items collected from all other areas. Black dots in **(b)** represent differences between mean numbers of items co-occurring with foods found more often in fresh scats and the mean number of co-occurring foods with these same items collected from unknown age scats. Values above the zero (the dotted line) indicate that more foods co-occurred with the more frequently consumed items, whereas those below indicate fewer co-occurred (or were excluded). Foods with 95% confidence limits that do not overlap zero (zero = no difference) indicate that polar bears consumed these foods at a relatively higher rate with significantly fewer (or more) co-occurring foods.

Four of the food items that were found to be spatially in excess of expectation also occurred with fewer accompanying food items compared to other areas, suggesting the bears consumed foods at higher rates in these areas and to the exclusion of other foods. This was the case with marine algae ( $\bar{x}$ = 2.02 ± 0.27 vs. $\bar{x}$ = 2.61 ± 0.14) in area #2, Lyme grass ( $\bar{x}$ = 2.31 ± 0.14 vs. $\bar{x}$ = 2.75 ± 0.25) and Lyme grass seed heads ( $\bar{x}$ = 2.05 ± 0.38 vs. $\bar{x}$ = 3.6 ± 1.42) in area #4, and berries ( $\bar{x}$ = 2.11 ± 0.34 vs. $\bar{x}$ = 4.00 ± 1.07) in area #5 (Figure 3a).

A total of 125 fresh scats with a total of 262 occurrences of food items was collected, all found along the coast. The proportions of foods from major categories (animals, vegetation and garbage) were significantly different (*G* = 6.30, *DF* = 2, *P* = 0.043) between fresh and unknown age scats due to an increase in the proportion of animals in fresh scats (*G* = 4.52, *DF* = 1, *P* = 0.0335). The frequencies of individual food items significantly differed (*G* = 36.79, *DF* = 13, *P* = 0.0004). More specifically, we found a higher proportion of Lyme grass seed heads ( $\hat{p}$ = 7.25, *CI* = 4.42-11.12 vs. $\hat{p}$ = 2.16, *CI* = 1.39-3.20) and lower proportions of marine algae ( $\hat{p}$ = 14.12, *CI* = 10.14-18.92 vs. $\hat{p}$ = 23.35, *CI* = 20.95-25.36) and berries ( $\hat{p}$ = 1.91, *CI* = 0.62-4.40 vs. $\hat{p}$ = 4.60, *CI* = 3.44-6.02) in fresh scats. Of these foods, only marine algae occurred in piles with significantly fewer accompanying foods in unknown age scats ( $\hat{p}$ = 1.69, *CI* = 1.56-1.83) than in fresh scats ( $\hat{p}$ = 2.84, *CI* = 2.45-3.23); and thus were consumed at higher rates to the exclusion of others later in the season (Figure 3b).

## Discussion

Climate-driven environmental changes are forcing polar bears to spend extended periods on land with smaller seal-based fat reserves. As such, land-based food consumed during this ice-free period may become increasingly important for survival and reproductive success [16,22]. The compositional and spatial patterns of these land-based foods can inform the extent to which terrestrial foraging may alleviate nutritional deficits associated with lost seal hunting opportunities. Currently, the polar bear diet on land is diverse, consisting of many plants and animals, often consumed together in various combinations. Even though they are consuming a mixed diet, polar bears consume higher rates of specific foods, sometimes to the exclusion of others, (e.g., Lyme grass seed heads, berries and marine algae) and often deposit these scats in areas where these foods occur most abundantly, suggesting little movement among habitat types between feeding sessions. The remains of animal prey were found often in scat but unlike plant material there was no obvious spatial pattern to their occurrence. In the following, we discuss possible reasons for the observed dietary patterns, how they may differ between sex and age groups and suggest potential benefits to polar bears consuming a mixed, omnivorous diet on land.

Foraging on vegetation was pervasive across the study area and certain plants were consumed more often, especially in areas where they predominated and where polar bears spent substantial time once they were ashore. Lyme grass, for example, occurs on primary and secondary beach ridges along most of the coast south of Cape Churchill to the Owl River (Figure 2, area #4) and is used extensively for temporary beds by arriving polar bears ( [33,49], unpublished observations). While lying in these beds, polar bears consume the entire Lyme grass plant (above ground parts), but will often preferentially consume just the seed heads [14,49] that mature in early to late July and remain available until late August [40].

The bears have also been seen walking through these stands of Lyme grass eating just the mature seed heads (unpublished observations). The relatively high gross energy yield (compared to other grains) [50], relatively high protein content [51] and convenient access would make these seed heads an attractive food source to arriving polar bears. It could also explain why many (44.1%) of the “fresh” scats contained seed heads and occurred with fewer accompanying foods where they were most abundant along the coast (area #4, Figure 2). They also comprised entire scats more often than any other food we recorded (39.5%; Figure 1).

Berries and marine algae were, similarly, found in scats more often where they predominated, but were likely consumed later in summer or early fall. Consistent with an earlier study [52], berry remains in scats were concentrated further inland, where mainly adult females with and without cubs as well as some subadults occur [53]. Berries were consumed more often and to the exclusion of other items, likely in late summer and early fall when commonly consumed species, such as “blueberries” (alpine bilberry, *Vaccinium uliginosum*) and black crowberry (*Empetrum nigrum*), ripen. During early fall, many polar bears congregate along the coast east of Churchill, where the landscape is dominated by Larch Fen and Bogs (area #2, Figure 2) [54], waiting for the ice to refreeze. Here, marine algae are more common than other vegetation and therefore may be more convenient to consume. Polar bears may also consume these plants at higher rates later in the season (and not when they first arrive onshore) to consume more desirable parts that become available in fall [55]. Also, shoreline piles of decaying marine algae often contain high concentrations of tipulid (cranefly) larvae (unpublished observations), which may attract polar bears to the plants later in the season when the insect larvae reach maximum size [56]. Reports of polar bears consuming marine algae in other regions, even when seals were available, have also been documented [57-60].

Animals, occurring in 45.8% of scats, are commonly consumed by polar bears during the ice-free period, however, we found no spatial patterns in scats containing them. One reason for this could be that because passage rates are longer for animals than plants [43,44], so they are moving between habitat types faster than the time required to defecate animal remains. Although given the small difference in passage rates (6–12 hours) and limited movements of polar bears on land [33,45], we feel it is more likely due to the widespread occurrence and/or mobile nature of the land-based animals that polar bears consume. For example, most of the birds consumed are various species of flightless waterfowl, the most common of which is Lesser snow geese, occurring in 12.5% of scats [16]. Since the 1960s, the population of snow geese in the Cape Churchill Peninsula has grown nearly 20-fold and expanded its nesting and brood rearing range from the La Pérouse Bay area to the entire Cape Churchill Peninsula as far south as Rupert Creek [23,61].

Earlier arriving polar bears have begun to overlap the incubation period of snow geese (and other waterfowl species) [23], but at present more commonly arrive while young and adult geese are flightless and dispersing along the coast to forage on graminoids. Similarly, the bears co-occur with caribou, whose numbers have increased substantially since the 1960s and that have expanded their summer range closer toward the coast [62], where interactions with arriving polar bears are common (unpublished observation). Other prey, such as rodents, are less mobile but are common in upland habitat, occurring within 5 km from the coast in years when they are abundant [63,64].

**Figure 4:**
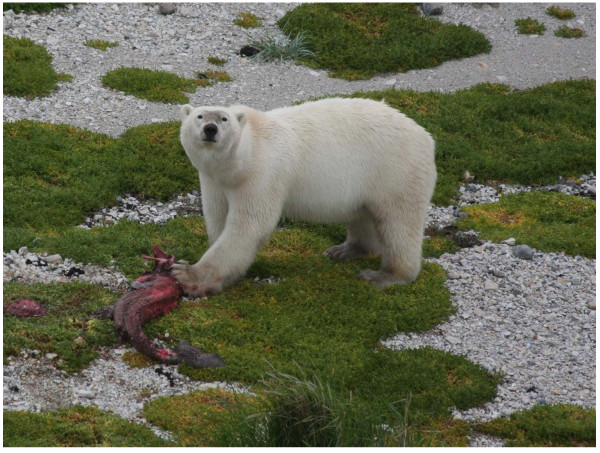
**A polar bear consuming a seal it captured during the ice-free season.** A polar bear guards the seal it captured and is consuming on the shore of Hudson Bay near the Seal River, north of Churchill, Manitoba, Canada, on August 14, 2010. Photograph by R. F. Rockwell.

Seal, being a preferred food, often occurred alone in scats and with fewer accompanying items. Although it is unclear whether seals were captured on the sea ice or from land (predation or as carrion), we observed multiple seal carcass remains on shore while sampling (unpublished observations). Further, when consumed with other foods, 57.1% of those were either land-based vegetation (i.e., grasses, moss, mushrooms; 21 of 42 scats) or land-based animals (i.e., birds, eggs; 9 of 42 scats). Others have similarly reported polar bears consuming seals and land-based food together through inspection of stomach contents, scat and direct observation [14,16,31,32,60,65]. The purpose of this diet mixing is unclear but could serve to dilute toxins accumulated in the flesh of seals [6,66-69]. Though capture of seals from the shore (e.g., seals resting on rocks) or in open water is considered rare [57], it does occur (Figure 4) ( [70], C.J. Jonkel pers. comm.) and may be responsible for some of the seal remains found in our study.

The spatial and compositional patterns in foods consumed suggest that polar bears are foraging on individual foods opportunistically. That is, they are consuming vegetation where and when it is most abundant and in random combinations with other foods (i.e., their occurrences are statistically independent) [16]. Despite the fact that polar bears are known to move little while on land [33,45], animal consumption appears to have no spatial component, likely because the animals are ubiquitously distributed and mobile. It is unclear whether foraging, in and of itself, is opportunistic or coincident with other observed behaviors. For example, is the intense foraging on Lyme grass coincident with resting on it once ashore or do they rest on Lyme grass after seeking it for consumption? Do certain demographic groups travel inland to consume more berries or are they consuming them coincident with being inland (where berries are more abundant) to avoid the threat of intraspecific predation along the coast?

Though the propensity to forage may differ between individuals, when it does occur, polar bears likely employ foraging tactics that minimize travel to conserve fat reserves. Polar bear locomotion on land is inefficient with costs of travel increasing with decreasing size [71,72]. As a result, search costs would be expensive, making it beneficial for polar bears to partake in large volumes of readily available food and include a large range of possible choices [5]. The generally high species richness and varied composition in scat that we observed, as well as apparent high consumption of locally abundant vegetation supports this. We also found that polar bears often consume more than one type of vegetation in a single feeding session (42.4% of scats). Switching between different types of plants may help maintain both high search efficiencies and bite rates, perhaps making longer bouts of foraging (with increased movements) energetically profitable [24,73].

Adopting a mixed diet of both animal-based protein and plant-based carbohydrates, which occurred frequently in our study (34.3% of scats), may allow polar bears to overcome some of the nutritional constraints associated with large body mass and inclusion of low quality forage in their diet. Other bear species are known to seasonally specialize on certain types of vegetation [73,74] but simultaneously consume animal-based protein and fat sources in limited amounts to maximize mass gain [9,12]. Robbins et al. [12] postulated that brown bears consumed an optimal combination of protein and carbohydrates that minimized the costs associated with protein digestion (deaminating and excreting excess nitrogen) while maximizing digestible energy intake. As a result, bears in the study gained disproportionately more mass on the optimal diet, than they would have gained from the same calories of each macronutrient alone. Polar bears on the Cape Churchill Peninsula may be optimizing their macronutrient intake during the ice-free season in a similar fashion.

Diet food mixing also has the potential to yield nutritional benefits beyond immediate mass gain. For example, various types of vegetation may provide vitamins and minerals absent from their primary diet [6,75]. Iversen [60], for example, describes specific vitamins and minerals in marine algae that are lacking in seal blubber, that might explain why polar bears of all sex and age classes (including adult males) consume this and terrestrial vegetation in Svalbard even when seals are still available to hunt. This may also explain observations of polar bears expending energy to dive, then selectively eating only specific parts of marine algae plants (C.J. Jonkel pers. comm., [14,57,58]).

Another non-energetic benefit of consuming a mixed diet is to allow sampling of available food to assess quality for potential switching or adding of new foods [5]. Traveling to new patches and the effort associated with capturing new prey (e.g., trial and error) are costly and may not yield an immediate energetic gain, however, greater familiarity with various food patches and improvement in efficiency of capturing prey may yield a net energy profit over an animal’s lifetime or that of its offspring [27,76,77]. A possible example of this would be the pursuit and capture of flightless waterfowl on land by polar bears. Although some report that consuming a goose after a long pursuit can not be energetically profitable [49], multiple observations of such behavior and the frequent occurrence of waterfowl remains in scat (28.0%; 180 of 642 scats) indicates successful captures occur often ( [27], unpublished observations). Given that geese are still a relatively new resource in western Hudson Bay [16] polar bears probably possess varying levels of expertise in capturing them. It may be that only until they have optimized their hunting technique will polar bears glean an energetic benefit from pursuing them.

The sex of polar bears consuming different foods can not be determined from our data without further genetic analyses, however, based on the tendency for different sex/age classes to segregate once ashore and move little on land [33,45,53], general inferences can be made. For example, females with cubs and sub-adults tend to move further inland, whereas adult males tend to predominate along the coast [53], which could lead to some partitioning of resources, as peak availability of certain foods (e.g., berries) might be more accessible to certain demographic groups. Derocher et al. [52] similarly noted the importance of broad spatial (and temporal) sampling in assessing the importance of terrestrial plants in the diet due to observations of berries being primarily consumed by adult females and sub-adults further inland. Although we found no spatial patterns in animal remains in scat, it is conceivable that travel to inland areas might increase interactions with more mobile prey, such as nesting waterfowl. Edwards et al. [4] reported that the degree of carnivory among female grizzly bears increased linearly with movement rate in the Mackenzie Delta region.

## Conclusions

Our results support previous findings and Traditional Knowledge that polar bears are opportunistic foragers that exploit a wide variety of plants and animals (e.g., [14,19]). There are clear spatial patterns of food use, especially among plants, and ample evidence that multiple different foods are consumed during single feeding sessions. These foraging patterns define food mixing and omnivory strategies on relatively small spatial and temporal scales. They would permit the bears to maximize calorie intake while minimizing energy expenditures associated with movement [12]. Non-energetic benefits, such as fulfilling vitamin/mineral requirements, diluting toxins, assessing new resources and learning processes, may also motivate seemingly unprofitable foraging behaviors [5,67,76,77].

We suggest that future research include genetic analyses to allow definition of the diet compositions of individuals of known identity and gender. That research should also establish the energetic costs of foraging to obtain mixed and omnivorous land-based diets as well as the energetic gains, including those obtained through food synergism, from those diets. Such information will allow the development of more realistic models of the effect of climate change on survival and reproductive success than current models that assume no nutritional input during the increasing ice-free period (e.g., [78,79]). Finally, future research should continue to monitor changes in polar bear foraging that may result from the bears responding to their changing environment.

## Authors’ contributions

Both LJG and RFR participated in the design of the study. LJG collected the data, identified and analyzed items in scat, performed statistical analyses and wrote the manuscript. RFR participated in the collection of data, assisted in the analysis of scat contents and statistical analysis, and edited drafts of this manuscript. Both authors read and approved the final manuscript.

## Supplementary Material

Additional file 1: Figure S1 Polar bear scat collection areas. Polar bear scat was collected along the coast of western Hudson Bay from the town of Churchill, Manitoba, to Rupert Creek. Scat was also collected near maternity dens at 6 inland sites. Collections were made from 2006 through 2008. Click here for file
