# Supplementary figures and images for: Dietary composition and spatial patterns of polar bear foraging on land in western Hudson Bay
Source: BMC Ecol. 2013 Dec 21;13:51. doi: 10.1186/1472-6785-13-51 (PMC3923008; doi:10.1186/1472-6785-13-51)

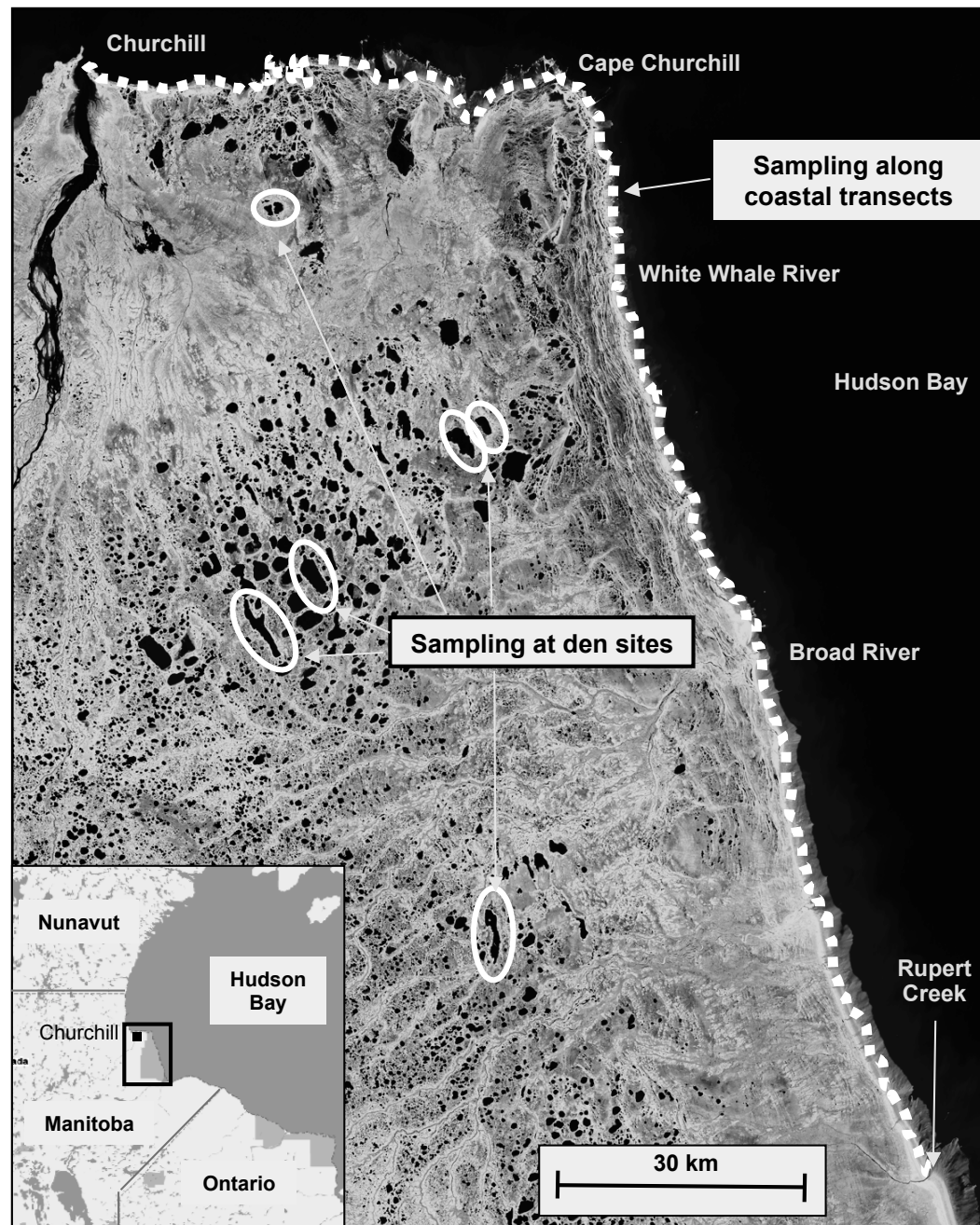

Figure A1

Supplement: Additional file 1: Figure S1 — Polar bear scat collection areas. Polar bear scat was collected along the coast of western Hudson Bay from the town of Churchill, Manitoba, to Rupert Creek. Scat was also collected near maternity dens at 6 inland sites. Collections were made from 2006 through 2008. [file 1472-6785-13-51-S1.pdf]
